# Developing a Public Health Course to Train Undergraduate Student Health Messengers to Address Vaccine Hesitancy in an American Indian Community

**DOI:** 10.3390/ijerph21101320

**Published:** 2024-10-04

**Authors:** Chassity Begay, Carmella B. Kahn, Tressica Johnson, Christopher J. Dickerson, Marissa Tutt, Amber-Rose Begay, Mark Bauer, Nicolette I. Teufel-Shone

**Affiliations:** 1 Center for Health Equity Research, Northern Arizona University, Flagstaff, AZ 86011, USA; marissa.tutt@nau.edu (M.T.); nicky.teufel@nau.edu (N.I.T.-S.); 2 College of Population Health, University of New Mexico, Albuquerque, NM 87131, USA; ckahn@salud.unm.edu; 3 Public Health Program, Diné College, Shiprock, NM 87420, USA; tresjohnson@dinecollege.edu (T.J.); cdickerson@dinecollege.edu (C.J.D.); ardbegay@dinecollege.edu (A.-R.B.); mcbauer@dinecollege.edu (M.B.)

**Keywords:** health messenger, COVID-19 pandemic, public health students, COVID-19 vaccine, Diné, health communication

## Abstract

The purpose of the Diné Teachings and Public Health Students Informing Peers and Relatives about Vaccine Education (RAVE) project was to develop strategies for health communication that addressed COVID-19 vaccine safety for residents of the Navajo Nation. The RAVE project developed a 16-week course using the Diné Educational Philosophy as a framework to train Diné College (DC) public health undergraduate students (*n* = 16) as health messengers to share COVID-19 vaccine safety information with unvaccinated peers and relatives. An online community survey (*n* = 50) was used to assess DC community vaccination perceptions to guide course development. The two primary reasons survey participants got vaccinated were to protect the health of others [82% (*n* = 41)] and to protect their own health [76% (*n* = 38)]. A pretest/post-test and a retrospective pretest (*n* = 13) were implemented to determine course effectiveness. A finding approaching significance was related to student confidence in being health messengers (9.1% increase). RAVE offers the first example in the published literature of successfully training American Indian undergraduate students in the context of a public health course to contribute to the response workforce during a public health crisis.

## 1. Introduction

American Indians and Alaska Natives (AI/ANs) were disproportionately affected by COVID-19, with higher COVID-19 incidence and mortality compared to the United States (US) population [1]. The Centers for Disease Control and Prevention (CDC) 2021 data showed that COVID-19 incidence rates for AI/ANs were almost four times that of non-Hispanic White Americans in 2020 [1]. Among AI/AN populations in the US, the Navajo Nation (NN) experienced the most COVID-19 cases early on in the pandemic, with 2304 positive cases per 100,000 residents, while the US rate was 636.3 positive cases per 100,000 residents [2]. The largest AI/AN tribe in the US, NN, has over 300,000 enrolled members with ~174,000 living on the over-27,000-square-mile reservation spanning parts of Arizona, New Mexico, and Utah [3]. Diné is how citizen members of the NN refer to themselves [4]; the terms Diné and Navajo are used interchangeably throughout this article.

The NN was among the first American Indian (AI) tribes to participate in the Pfizer/BioNTech COVID-19 vaccine clinical trial. The NN leaders within the NN government hosted a town hall forum via social media to increase vaccine confidence and address vaccine concerns among NN citizens [5]. To mitigate COVID-19 incidence, vaccination sites opened throughout the NN in Indian Health Service (IHS) facilities and tribally controlled high schools. In December 2021, 71% of eligible NN residents were fully vaccinated when the US vaccination rate was 60% for adults during the same period [6,7]. Despite the high vaccination rate and strong public health efforts, reducing COVID-19 vaccine hesitancy remains a challenge for the NN and other AI/AN populations.

Throughout the pandemic, vaccine hesitancy among the US population created concerns about achieving population or “herd” immunity [8]. Vaccine refusal among AI/AN communities was driven by past and current inequities created by historical trauma, colonization, mistrust, and social disadvantage [9,10]. Further challenges, such as limited access to electricity, water, and adequate healthcare services, collectively contributed to an increased risk of COVID-19 [11]. Despite facing many challenges related to the pandemic, AI/AN communities continuously showed how connection to culture, land, language, and community provided the ability to overcome hardships and difficulties [12,13,14].

At the time of reporting, no published literature describing the training of AI/AN college students as health messengers through a public health course was identified. The available literature on non-AI/AN college students indicates they were trained as peer health educators for other college students to provide health-related education as a prevention strategy [15]. College-level peer health educators have demonstrated efficiency in promoting health-related content and have been effective in shaping attitudes, values, and behavior [16]. Dubovi and Sheu [16] developed and tested the effectiveness of a peer health educator training program to improve self-efficacy and promote health-related outcomes among peers. In this context, peer educators showed an improvement in personal growth.

The Navajo NARCH project has the goal of creating a pathway program to build a public health workforce on NN [17,18]. As of November 2021, nationwide, only 24% of AI/ANs were enrolled in college compared to 41% of the overall US population [19]. Data from June 2022 show that 20.6% of US graduates earned master’s degrees though only 0.5% of those graduates were AI/AN [20]. Tribal college public health courses offer a way of strengthening the pathway to train more AI/AN public health messengers and to expose emerging public health professionals to the value of culturally tailored messaging and the role of research and evaluation to guide effective programming.

To address vaccine hesitancy, the Diné Teachings and Public Health Students Informing Peers and Relatives about Vaccine Education (RAVE) course was designed to develop strategies for health communication addressing COVID-19 vaccine safety for NN residents. RAVE was a supplemental project awarded by the National Institute of Health’s (NIH) National Institute of General Medical Sciences (NIGMS) to the Navajo Native American Research Center for Health (NARCH)—a partnership between the Diné College (DC) public health program [21] and the Center for Health Equity Research at Northern Arizona University (NAU) [17]. In addition to providing public health support, the students on the RAVE course had an opportunity to learn about the roles of health messengers and gain interest in future careers involving health communication or community outreach.

Current Diné educators express the need to use Diné-centered pedagogy when creating educational courses for Diné students [22]. The pedagogy for the RAVE public health course applied the Diné Educational Philosophy (DEP) as the overall framework to develop health message materials and teach students to engage community members using a Diné worldview. Other educational models in the course included Diné pedagogy centered on teachings and concepts based on *k’é* (relationships) and Sa’ah Naaghái Bik’eh Hózhóón [22]. Four Diné principles from the philosophy of Sa’ah Naaghái Bik’eh Hózhóón (e.g., don’t be lazy, use all of your strength and capacities, it’s up to you, and believe in what you are doing) articulate the course lessons developed for students that encompassed Diné way of life, identity, and inherent strengths [22]. The active learning aspects of the course required students to recruit participants and conduct individual health messenger sessions.

The purpose of the RAVE project was to train DC undergraduate public health students as health messengers to address COVID-19 vaccine hesitancy on NN. An online community survey for DC employees and students was used to understand the perception of COVID-19 vaccination and inform course development. This work is grounded in Hooper et al.’s [23] findings that education tools tailored to community and cultural characteristics are more effective approaches to health communication and education than standard methods among AI/AN populations. This paper will discuss the methods and results from the DC online community survey used to inform course development, the RAVE course content, and the RAVE course pre-/post-test and retrospective pretest.

## 2. Methods

All data collection methods were approved by the Navajo Nation Human Research Review Board, the DC Institutional Review Board (IRB), and the NAU IRB. The Navajo NARCH Community Advisory Board was used to give feedback on the overall RAVE course design and offered input on how the project and health message materials could be disseminated. The NARCH Community Advisory Board consists of public health and educational professionals from the NN, as well as NARCH research team members from NAU and DC. The board met each month on Zoom (San Jose, CA, USA) to hear monthly updates regarding the project. The RAVE project created an online consent and online community survey distributed to DC students, faculty, and staff. Consent was also obtained from both the DC students enrolled in the RAVE course as well as the peer and relative participants in the educational sessions. The RAVE course was developed and offered weekly to train undergraduate students as health messengers.

### 2.1. DC Online Community Survey Development

Prior to course development, RAVE implemented an online survey of community knowledge and attitudes on COVID-19 for DC students, faculty, and staff of 18 years and older who worked or attended school in-person or remotely across all DC sites. This population was surveyed because these respondents would provide some of the nuances of vaccine hesitation that would need to be included in the health messenger training. The online community survey assessed COVID-19 vaccination status and vaccination motivation and hesitancy among the respondents, their peers, and relatives, as well as strategies used to access COVID-19-related information. The 30-question survey, consisting of 13 demographic questions and 17 COVID-19 and vaccine-related questions, was delivered via SurveyMonkey (San Mateo, CA, USA). Recruitment occurred through a mass email listserv containing 1315 individuals and through a post on the DC Facebook page with the survey link. Information about the purpose and risks of the survey were included on the first page, and respondents were informed that they were providing passive consent to participate by clicking ‘next’. The survey was anonymous and had no incentive, and the survey invitation was sent by mass email three times during the month it was open, leading to a self-selected sample.

For the DC online community survey, data were collected and maintained using a password-protected account on an online survey platform and then downloaded for analysis as needed. Descriptive statistics and bivariate comparisons of answers were completed. The questionnaire was not validated due to the nature of the pandemic and the need for immediate interventions.

### 2.2. Diné Educational Philosophy (DEP) Framework

At DC, the DEP is the grounding framework for all educational programs to situate student learning within the context of Diné values, knowledge, and teachings. The philosophy explains the Diné value of living a full and complete life through harmony and balance with the natural world [21,24]. The philosophy encompasses early thinking (newborn stage), planning (adolescent stage), living (adulthood), and disseminating knowledge (elderly stage). By using this framework, students understand the relationality of Diné teachings with Western-based concepts of public health. The Diné teachings come from the philosophy of Sa’ah Naagháí Bik’eh Hózhóón, which encompasses the teachings on how to live in order to reach old age [22]. The key concepts and values in the DEP are sequential and can be expressed with the following Diné words: Nitsáhákees (thinking), Nahat’á (planning), Iiná (living), and Siih hasin (synthesis of knowledge).

### 2.3. RAVE Course Development

The DEP framework aligns with RAVE’s goals and guides each step of the research process. Additionally, the DEP includes a wellness perspective that also values *k’é*, a principle of how Diné are connected or related through clanship, and provides mutual respect when working together as relatives to consider options and identify solutions [25,26]. Guided by *k’é* (relationships), the project goals were to empower students with cultural and scientific knowledge to support their peers’ and relatives’ decision-making about vaccine acceptance. Following this and other Diné teachings, the course presented a view of public health focused not primarily on problems (i.e., statistics indicating Diné have high rates of a certain disease or condition) but on positive aspects of Diné life and health as well as ways in which those positive aspects can be celebrated and strengthened. Relatedly, cultural assets tied to Diné health-related teachings provide a cultural foundation for public health’s breadth and depth. The RAVE course was inspired by the vision of the public health workforce improving the cultural relevance and effectiveness of public health programs serving the Diné people and potentially other AI/AN groups.

**Figure 1:**
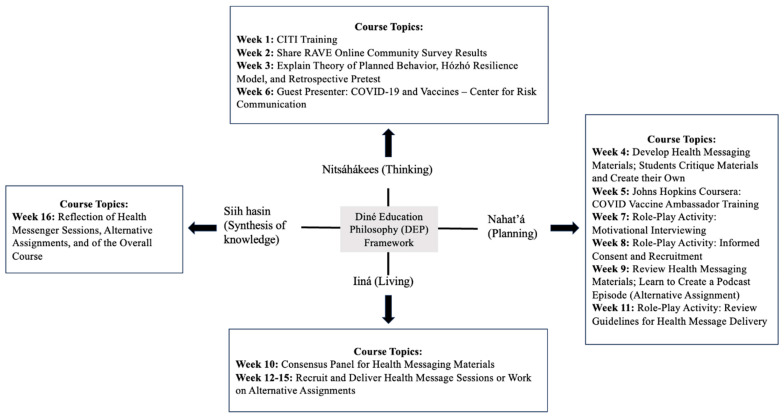
DEP framework for RAVE course development.

The DEP provided the framework for creating a culturally responsive teaching environment allowing students to engage in an Indigenous-focused course while contributing their knowledge, values, and experiences to the health message materials. The course used a unique pedagogical approach for Diné public health education by developing the course topics for undergraduates through the DEP model (Figure 1). For the thinking stage (Nitsáhákees), the curriculum first focused on building student knowledge and self-confidence in understanding the process of creating health message materials as well as learning research concepts and health behavior theories and models from both Western and Indigenous contexts.

In the planning stage (Nahat’á), the curriculum included active learning activities to prepare students for their roles as health messengers through practice and skills in areas such as effectively communicating, critiquing health message materials, and learning motivational interviewing (MI). Course time was used for students to interact with each other and practice with partners in breakout rooms on Zoom. Students applied critical thinking by including their own knowledge and skills into the curriculum and incorporating Indigenous worldviews and language into the materials.

In the DEP living stage (Iiná), the curriculum supported students to experientially apply their health messenger skills in a community setting and use a co-learning approach with their peers and relatives through MI. The teachings of *k’é* also helped students feel confident in reaching out to peers and relatives they cared for while creating an opportunity to engage them in understanding their concerns about being vaccinated.

The synthesis of the knowledge stage (Siih hasin) of the curriculum supported students in reflecting on their roles as health messengers including authentic learning to understand the real-world application of health messengers in community-engaged settings. The students provided an in-depth assessment of the course through discussion, a course evaluation form, and a retrospective pretest to offer feedback on their experiences and suggestions on how the course could be improved.

The results from the DC online community survey highlighted important areas of DC students, faculty, and staff’s perceptions of vaccines and COVID-19 that were included as topics in the course. The survey indicated that a high number of respondents were hesitant, so the course instructors elected to use motivational interviewing (MI) as a way to help apprehensive students understand their role as health messengers. The students felt more confident knowing their role was not to persuade others but to understand others’ hesitancy while sharing health information to address questions or gaps in knowledge about the vaccines. The survey also provided insight into the diverse opinions of the COVID-19 vaccine and the high level of hesitancy, which helped determine the need to provide more relatable health message materials that may be relatable from both Western science and Indigenous perspectives. In addition, the survey indicated that respondents were obtaining COVID-19 vaccine information from a variety of media channels including social media and the radio. These results helped shape the direction of the course materials through handouts that could be shared as a hardcopy or by computer, a digital story that was visual, and a podcast episode available online.

### 2.4. RAVE Course

In December 2021, a one-unit, special topics, public health course was developed as part of the RAVE project to train students as health messengers. There were no criteria for students to enroll in the course. The lead instructor was a Diné DC faculty member with a Diné NAU graduate teaching assistant. The 16-week class, for which students received a $500 stipend for completing, met remotely, via Zoom, for one hour once a week with 11 weeks of didactic training, four weeks to deliver educational sessions, and one week for guided student reflections and analysis of education outcomes.

Sixteen (16) DC students were trained to be health messengers through interactive education and practice to deliver credible, vaccine safety information. Students were tasked with recruiting, consenting, and delivering the education materials. Students were tasked with recruiting at least five adult peers or relatives (≥18 years of age) who were unvaccinated and lived or worked on NN. The students and peer or relative participants did not need to self-identify as Diné or AI/AN. The students had a unique role in RAVE by leveraging their academic training and social and familial relationships to educate NN residents about vaccine safety. The research team and Diné community health representatives (CHRs) [27] developed the health message materials, described by Tutt et al. [28]. The students shared the materials with up to five participants per session and were trained to use MI techniques [29] to ascertain perceptions of the COVID-19 vaccine and reasons for vaccine hesitancy. The students used a course developed, health messenger guide to assist with asking questions and delivering the education.

The course evaluation consisted of a 12-question pretest and a 28-question post-test administered to students to assess their experience as health messengers and as participants in the RAVE course. The pretest asked about demographic information and four questions on knowledge and confidence surrounding health messaging or COVID-19 vaccines. The post-test repeated the four questions on knowledge and confidence and asked 21 questions related to course evaluation.

The course also included a retrospective pretest in addition to the course post-test to assess changes in the students’ knowledge and perceptions of being a health messenger. The retrospective pretest asked students first to report on their current knowledge and perceptions and then to complete the same self-report measure with reference to where they perceived themselves prior to the course [30]. The three-question retrospective pretest compared pretest answers when students had limited information on health messaging for COVID-19 vaccines with how students would have answered if they were already informed about health messaging for COVID-19 vaccines to determine changes in knowledge and confidence for being a health messenger. The course evaluation was collected through an online survey platform and downloaded for analysis. Responses were collated in Excel and then descriptive statistics and Chi-square analysis were completed as determined by outcome–response distribution using OpenEpi.com (accessed on 30 November 2022) [31].

## 3. Results

### 3.1. DC Online Community Survey

A convenience sample of 50 respondents completed the online community survey. Table 1 demonstrates the demographic information from the sample. The count of question responses as response rates varied by question. The online community survey was available to DC students, faculty, and staff who were 18 years or older and worked or attended school in-person or remotely. Most respondents were online students and were geographically distributed across the NN, though 10% (*n* = 5) resided off the reservation or at other locations across the US. When asked about vaccination status and reason for getting vaccinated, 96% (n = 48) had been at least partially vaccinated, with 47 indicating being fully vaccinated. When given the opportunity to provide multiple responses, 82% of respondents identified wanting to protect the health of others as their primary reason for getting vaccinated (n = 41). Other responses included protecting their health [76% (*n* = 38)], being scared of contracting COVID-19 [46% (*n* = 23)], and being required by an employer [42% (*n* = 21)]. No one got vaccinated to receive an incentive.

Of the 50 respondents, 56% (*n* = 28) agreed that vaccine safety was a concern, while 18% (*n* = 9) were neutral, and 26% (*n* = 13) disagreed. Hesitancy was more evenly split between being hesitant, 42% (*n* = 21), and not being hesitant, 36% (*n* = 18), with the remainder neutral. When asked how many of their relatives seemed hesitant, the results were evenly split between “many/some” and “few/none.” Reasons for relatives’ hesitancy identified doubts about safety, 66% (*n* = 33), and concern about possible side effects, 66% (*n* = 33). Additional reasons included doubts about the seriousness of the virus 38% (*n* = 19), doubts about the likelihood of exposure, 30% (*n* = 15), and being warned about the vaccine by family or friends, 20% (*n* = 10), or political leaders, 18% (*n* = 9). Peers were perceived to exhibit less vaccine hesitancy with “few/none”, 64% (*n* = 32), outpacing “many/some”, 36% (*n* = 18). Reasons for peers’ hesitancy included doubts about safety 64% (*n* = 32), possible side effects, 58% (n = 29), doubts about the seriousness of the virus, 32% (*n* = 16), the likelihood of being exposed, 26% (*n* = 13), and warned by family or friends, 22% (*n* = 11), or political leaders, 16% (*n* = 8). Few (<7) relatives or peers believed getting vaccinated was against cultural or religious teachings.

Respondents were asked if they previously had COVID-19 as well as how many of their relatives and peers had been infected and how many had died. Ten (20%) respondents previously had COVID-19, while the average number of reported family members infected was 9.19 (SD 7.44) and that of peers infected was 4.9 (SD 4.72). The average number of family members who died was 1.81 (SD 2.9) and that of peers who died was 1.16 (SD 2.04).

Given concerns about widespread misinformation through the media regarding COVID-19 vaccination, respondents were asked where their vaccine-hesitant peers and relatives received their information (Table 2.) Respondents were asked whether their vaccine-hesitant peers and relatives followed the news closely: 56% (*n* = 28) agreed they did and 42% (*n* = 21) disagreed. The influence of politics was also of interest; therefore, for perceptions of whether vaccine-hesitant peers and relatives followed politics closely, 44% (n = 22) agreed, 16% (n = 8) disagreed, and 38% (n = 19) responded neutrally. Respondents noted that their vaccine-hesitant peers and relatives received most of their information from Facebook, 60% (*n* = 30), followed by local television, 54% (*n* = 27), radio, 32% (n = 16), local newspapers, 24% (n = 12), and unsure, 24% (n = 12). Ten (20%) or fewer respondents identified their sources as national newspapers, cable news, and other. 

Since the purpose of the DC online survey was to inform course development, patterns were examined but tests of significance were not performed. Doubts about vaccine safety, the likelihood of being exposed to COVID-19, and being concerned about side effects occurred more among individuals who indicated not being unconcerned about vaccine safety, which was counterintuitive. Respondents who self-identified as hesitant had doubts about vaccine safety, the likelihood of exposure, and the seriousness of the virus as well as possible side effects. Respondents perceived the barriers for their vaccine-hesitant relatives were related to having doubts about vaccine safety and being warned against the vaccine by family or peers. Interestingly, respondents said that their peers’ reasons for vaccine hesitancy were related to the seriousness of the virus.

### 3.2. Student Health Messaging Education

To determine the effectiveness of the course as an educational intervention, students completed a pretest/post-test with a retrospective pretest given alongside the post-test. All responses were on a five-point Likert-type scale ranging from “Strongly Agree” to “Strongly Disagree” which were collapsed to “Agree” or “Neutral/Disagree” for analysis. The students showed improvement in the desired direction though not always at a statistically significant level.

The course had a 35-student capacity but only 16 students were enrolled in the course. Due to various levels of student engagement, 81% (*n* = 13) of students completed both portions of testing for the analysis. Of the 81% (*n* = 13) who completed the assessment, approximately 75% identified as Diné, 75% were female, 85% were public health majors, and 38% indicated having previous experience delivering health messages. The course sex ratio was in line with the college ratio [21]. The participating Diné public health majors, freshmen through seniors, ranged in age from 22 to 62 years. From the pretest to the post-test (Table 3), student indication of understanding the background of COVID-19 increased by 50%, understanding of at least one vaccine increased by 100%, and confidence in the ability to deliver health messages increased to 20%. Due to the small sample size, only the change in vaccine understanding was statistically significant (*p* = 0.015). Students expressing confidence in the ability to make a difference by sharing COVID-19 health messages increased by 9%. The results showed that the students may have been overconfident in their understanding of COVID-19 (−12.5% change from the pretest to the retrospective pretest) and their ability to deliver health messages (−30%). However, the students may have had more knowledge of the vaccines than they initially realized (33.3%) but, as above, no changes were statistically significant.

## 4. Discussion

A key finding from the DC online community survey was related to information sharing. DC students and employees indicated that their vaccine-hesitant peers and relatives received most of their information primarily from Facebook and local television. Misinformation may be shared on social media platforms and can create hesitancy or concerns about vaccines [32]. To combat widespread misinformation sharing, more materials that are credible and relevant to the local populations could be created to address common misconceptions about vaccines. This online survey was valuable in not only highlighting the motivation for vaccine uptake but also identifying from what type of media sources the community members were obtaining their information.

The RAVE course designed for college students offered an innovative solution to train trusted messengers with relational ties to the recipients to increase knowledge of COVID-19 and the available vaccines within the community. Training public health college students as health messengers can contribute to the pool of existing health messengers, such as physicians, nurses, and CHRs, to address public health-related issues, particularly in a time of public health crisis. Health messengers can expand community-based health approaches and aid in relationship-building within communities that may lack long-lasting infrastructure in public health [32]. As lay health messengers, the students’ primary role was education and not patient navigation.

Using the DEP, course instructors centered sessions on Diné ways of knowing, lifeways, and language, the course content was informed by the DC online survey which was designed to understand lived experiences with the COVID-19 vaccines. Stories and knowledge from traditional knowledge holders [13] were included in the materials as a way to share spiritual education, Diné origin stories, and Diné teachings for understanding COVID-19 vaccines. Overall, the course was based on the teachings of hózhó and Sa’ah Naaghái Bik’eh Hózhóón [22] to help communities be in harmony and balance while facing COVID-19. The traditional knowledge holders also expressed in their interviews a desire to share their knowledge to support the students in learning about education in relation to health from a Diné worldview.

### 4.1. Diné-Centered Pedagogy

Applying a Diné-centered pedagogy for the RAVE course encouraged students to draw on their innate strengths and identities to complete their health messenger training and projects. The students used their cultural capacity and knowledge to help the course instructors create culturally relevant materials. The course was implemented online during the pandemic, and students demonstrated emotional capacity and strength when expressing the desire to gain knowledge about COVID-19 and vaccines to help their communities.

The course provided the opportunity to observe and complete in-class experiential assignments where students applied their training in authentic experiences before going into the community to share the health message materials and apply critical thinking when participants asked questions. The students received an in-depth understanding of the research process by completing human subjects training, learning the process for providing informed consent, and learning the data collection process.

An exchange of reciprocity occurred when students offered their knowledge and skills to peers and relatives, and in turn, peers and relatives allowed time for the sessions and shared information about their beliefs and perceptions about COVID-19 and vaccines. Students gained knowledge of community perspectives on vaccine hesitancy and reflected on that knowledge. The educational system is part of *k’é* and can be used as a pedagogy for relationships to promote student growth and success.

Some key lessons learned from the course indicate the need for flexibility with the course design and implementing a course curriculum that builds student confidence and emphasizes cultural strengths. The course was offered online through Zoom and was open to all DC students, even if they did not live on NN; therefore, course instructors had to be flexible and provide alternative assignments when students were unable to recruit the required number of participants.

Additional key points for student learning were course instructors teaching MI as a primary strategy to use when interacting with their peers and relatives and the application of *k’é*. The students stated that using MI techniques gave them the confidence to share health information and ask questions respectfully rather than feel like they were forcing information or trying to be persuasive. In the course, *k’é*, a Diné concept that stresses the importance of relationships, was seen as a cultural strength because students shared how their peers and relatives mentioned they were appreciative of receiving health information from a trusted close friend or family member rather than from health professionals with whom they felt no connection.

### 4.2. Limitations

The online community survey had a small sample size, so a simple, descriptive analysis was performed. Another consideration is the fact that the sample only included DC students and employees; therefore, the results reflect that special population and not NN residents generally. In addition, several respondents indicated that they were not living on NN when they took the survey.

A primary limitation of the RAVE course was the challenge students faced in recruiting at least five or more unvaccinated peers or relatives; most of the eligible adults they knew were vaccinated. This challenge may reflect that students interested in public health have peers and relatives receptive to health-protective services. The project focused on finding participants who were not fully vaccinated with their first and second dose. The eligibility criteria specified that students could not recruit participants who only needed booster shots. Of the 16 students who completed the course, seven students completed alternative assignments as they were unable to recruit five peers or relatives who were unvaccinated. Another limitation was the lack of time to train the students. The class met for one hour a week; thus, the students had limited time to practice MI or role-play for the educational sessions with peers or relatives. Lastly, memory-related biases from the retrospective pretest were not considered a limitation as the test defined the time period for students to recall their knowledge and perceptions; advocates of this method indicate that memory-related biases are introduced when respondents are asked to specify a date or time period [30]. Finally, within the constraints of the COVID-19 pandemic, there was no opportunity to plan and execute a research design with a control group for comparison; therefore, a causal relationship could not be established.

## 5. Conclusions

The overall RAVE course design included a culturally-based approach to train students by using the DEP framework and experiential activities to engage students. Teaching AI/AN students to become health messengers through Diné teachings and worldview can be an important strategy to increase trust and one’s ability to influence behavior change on health-related issues within tribal communities. A recommended strategy for other public health courses is to support AI/AN students in learning how to culturally adapt health education materials beyond Western contexts to ensure that health messages are more relevant to peers and relatives within their respective communities. Utilizing culturally adapted materials enhances and provides cultural relevance to recognize factors such as language and concepts of spirituality that impact health outcomes for AI/ANs [33]. The Diné-centered pedagogy helped students start their training with critical thinking, set goals and plans for creating the materials and recruiting and practicing, carry out their projects, and then complete self-reflections and be aware of the larger concepts after data analysis and learning from each other.

In addition to the culturally adapted materials, RAVE provided further insight into training health messengers who are knowledgeable and credible. Though there were few statistically significant results, the trends suggest that this approach increased the number of health-informed AI/AN communities to follow tribal government protocols during an outbreak or future pandemics [34]. In addition, the project was unique in the audience the health messengers reached. Health messengers recruited peers and relatives who resided or worked on NN, were unvaccinated, and had concerns or were hesitant to receive the vaccine. The health messengers understood the reasons and factors why community members were hesitant to receive the vaccine as most health messengers were AI/AN and from the same community as their peers and relatives. This approach to health communication recognizes how community is essential to AI/AN belief systems and should be implemented in college settings [35]. Overall, AI/AN college students have the potential to serve as a valuable and sustainable resource to educate community members on public health-related issues. A similar approach should be tested with other communities who have had negative experiences with Western medicine and thus may distrust treatment or prevention recommendations. The RAVE framework demonstrates a successful way to communicate vaccine education in an AI population and may show similar results in other culturally distinct populations.

## Figures and Tables

**Table 1:** DC online community survey.

Participant Demographics (N = 50)				
Age	18–24: 9	25–44: 27	45–54: 8	55+: 5
Gender	Female: 42 Male: 7 Other: 1			
A DC student	Yes: 31		No: 19	
Employed at DC	Yes: 19		No: 30	
Highest level of education	HS: 3 Some College: 14 AA/AS: 16 BA/BS: 12 Grad Sch: 5			
Hispanic or Latino	Yes: 3		No: 47	
Racial Category	Native American: 50		White/Caucasian: 2	
Live with individuals over 60 *	Yes: 13		No: 37	
Live with children under 18 *	Yes: 30		No: 20	
* Three participants lived both with individuals over 60 and children under 18				
**Count of Question Responses**				
Occupation				50
Employment status (check all that apply)				59
Tribal affiliation				47
City and state of residence				48
Received one shot of a COVID-19 vaccine				50
Fully vaccinated				49
Reason for vaccination (check all that apply)				167
Concerned about Covid-19 vaccine safety				50
Hesitant about the COVID-19 vaccine				49
Respondent had COVID-19				50
(None, few, some, many) relatives seem COVID-19 vaccine hesitant				50
Reasons relatives expressed for COVID-19 vaccine hesitance (check all that apply)				137
(None, few, some, many) peers seem COVID-19 vaccine hesitant				49
Reasons peers expressed COVID-19 vaccine hesitance (check all that apply)				128
Number of close relatives who had COVID-19				50
Number of peers who had COVID-20				50
Number of close relatives who died from COVID-19				50
Number of peers who died from COVID-20				50
News engagement of vaccine-hesitant peers and relatives				49
News sources of vaccine-hesitant peers and relatives (check all that apply)				123
Political engagement of vaccine-hesitant peers and relatives				49

**Table 2:** Peers’ and relatives’ information attentiveness and sources (n = 50).

Follow the News Closely		News Source	
**Agreed**	**56% (28)**	Facebook	60% (30)
Disagreed	42% (21)	Local Television	54% (27)
**Follow Politics Closely**		Radio	32% (16)
Agreed	44% (22)	Local Newspapers	24% (12)
Disagreed	16% (8)	Unsure	24% (12)
Neutral	38% (19)	National Sources	<20% (<10)

**Table 3:** Changes in students’ health messaging knowledge and confidence (n = 13).

Question	Pretest	Post	Change	*p*-Value	Pretest	Retro-Spective Pretest	Change	*p*-Value
I understand the background of COVID-19 well enough to explain it to my family members and friends.	61.54%	92.31%	50.00%	0.080	61.54%	53.85%	−12.50%	0.50
I understand at least one COVID-19 vaccine well enough to explain how it works to my family members and friends.	46.15%	92.37%	100.00%	0.015	46.15%	61.54%	33.33%	0.348
I feel confident to share health messages about COVID-19 and vaccines with my family members and friends.	76.92%	92.31%	20.00%	0.297	76.92%	53.85%	−30.00%	0.206
I feel I could make a difference in the health of my family members and friends by sharing the knowledge I currently have about COVID-19 and vaccines.	84.62%	92.31%	9.10%	0.5				

## Data Availability

The data presented are not readily available because the data belong to the Navajo Nation, according to the Navajo Research Act and longstanding IRB policy; therefore, any data sharing would have to be specifically approved by them, not by the authors. The dataset is small and includes details that could potentially reveal the identity of individual subjects. Requests to access the data should be directed to Dr. Nicolette Teufel-Shone at Nicky.Teufel@nau.edu.
